# Leprosy in a Patient With Lymphoma: A Challenge in the Twenty-First Century

**DOI:** 10.7759/cureus.50007

**Published:** 2023-12-05

**Authors:** Eulália Antunes, Inês M Araújo, Francisco Cubal, José L Sousa, Sueila Martins, Fernando Guimarães, Rita Tenreiro, Marisol Guerra, Manuel Cunha

**Affiliations:** 1 Internal Medicine, Hospital de Braga, Braga, PRT; 2 Hematology, Centro Hospitalar de Trás-os-Montes e Alto Douro, Vila Real, PRT; 3 Infectious Disease, Centro Hospitalar de Trás-os-Montes e Alto Douro, Vila Real, PRT; 4 Internal Medicine, Centro Hospitalar de Trás-os-Montes e Alto Douro, Vila Real, PRT

**Keywords:** rituximab, mantle cell lymphoma, relapse, hansen’s disease, lepromatous leprosy

## Abstract

Leprosy, or Hansen’s disease, mistakenly considered a disease from the past by some, is still common nowadays, especially in tropical and subtropical regions. In the absence of appropriate medical treatment, it may progress and cause permanent damage to multiple organs.

This case report illustrates the diagnostic challenge of a south-american adult man who had been living in Europe for over 14 years. He was referred to the Hematology department due to persistent lymphocytosis and a CD5+ B-cell lymphoproliferative disorder was identified. During clinical surveillance, the patient developed skin lesions in his limbs with associated hypoesthesia. A histological diagnosis of lepromatous leprosy was made, and he underwent a long-term three-drug therapeutic regimen (dapsone, rifampicin, and clofazimine). Adding to the complexity of the case, the patient progressed with splenomegaly and constitutional symptoms, more than 7 years after development of lymphocytosis. Through a comprehensive evaluation, a definitive diagnosis of mantle cell lymphoma was established and received 6-cycle R-CHOP induction, followed by maintenance rituximab. Importantly, prophylaxis for leprosy reactivation was not administered as there were no recommendations in available guidelines. Eventually, the patient experienced a leprosy relapse while on maintenance therapy, 58 months after completing the initial anti-leprous treatment. Clinical response was attained with a new treatment regimen consisting of rifampicin, clofazimine, and minocycline.

Although leprosy is primarily observed in tropical and subtropical regions, the long incubation period of this disease combined with the global flow of migrants, made us consider it. Despite being rare, leprosy relapses can occur even after a few decades. The contribution of rituximab or previously administered chemotherapeutic agents is still unknown. The question remains whether antibiotic prophylaxis should be performed in patients undergoing immunochemotherapy for malignant diseases.

## Introduction

Leprosy, also known as Hansen’s disease, is a chronic bacterial infection caused by *Mycobacterium leprae* and *Mycobacterium lepromatosis*. These acid-fast, gram-positive intracellular bacilli belong to the *Mycobacterium leprae* complex [1]. They primarily affect phagocytes in the skin and Schwann cells within peripheral nerves [2].

In 1990, the World Health Organization (WHO) proposed a global goal of eliminating leprosy by the end of the 20th century [2]. Although leprosy is currently rare in Europe, disease control has not yet been achieved in tropical countries, especially in resource-limited settings [3]. This is particularly concerning in Southeast Asia, America, Africa, the Eastern Pacific, and the Western Mediterranean [2].

Leprosy predominantly affects males, and the initial clinical manifestation often involves skin lesions [3]. Diagnostic confirmation is often achieved through skin biopsy, which can also help exclude other potential causes. To minimize the likelihood of resistance, the WHO has recommended a multidrug therapy regimen [4]. Without appropriate medical treatment, leprosy can progress and result in permanent organ damage [2].

We present a complex diagnostic approach to a patient with coexisting leprosy and mantle cell lymphoma, as well as the treatment protocols and follow up, which allowed the detection of a rare case of leprosy relapse after chemoimmunotherapy use.

## Case presentation

A 60-year-old man, born in Brazil and living in Portugal since the age of 47, with no relevant comorbidities except for arterial hypertension managed with valsartan 80 mg once daily, was referred to the Hematology department after detection of lymphocytosis in routine blood analysis. He denied experiencing weight loss, asthenia, fever, or night sweats, and there was no evidence of lymphadenopathy, palpable masses, or organomegaly in the physical exam. Laboratory workup confirmed mild lymphocytosis of 8.8x10^9/L (Table 1), with a predominant B-cell population of small-sized cells with abnormal immunophenotypic characteristics: CD19+, CD20+, CD5+, CD10-, CD200-, FMC7-, CD79b+, CD11c-, CD103-, CD38-, membrane Igs+ (IgM, IgD, kappa light chains) and inconclusive CD23 and CD25. Contrast-enhanced thoraco-abdominopelvic computed tomography (CT) revealed multiple dispersed lymphadenopathies in various mediastinal compartments as well as in azygo-esophageal recess, with a maximum short axis of 6 millimeters (mm), and several scattered throughout the retroperitoneal mesentery, with a maximum short axis of 7 mm; spleen had normal size and morphology, and there was a small accessory spleen measuring 11 mm. Thus, a CD5+ B-cell lymphoproliferative disorder was identified, with inconclusive phenotypic features for further classification. Given the absence of treatment criteria at that time, clinical and analytical surveillance was initiated.

**Table 1:** Laboratory workup at the first hematology consultation evaluation and seven years later (time of mantle cell lymphoma diagnosis).

	First hematology evaluation	Seven years later
Hemoglobin (11.9-15.6 g/dL)	13.5 g/dL	10.9 g/dL
Hematocrit (43-55%)	42.4%	32.7%
Mean corpuscular volume (87-103 fL)	88 fL	74 fL
Mean corpuscular hemoglobin (27-33 pg)	28.7 pg	24.8 pg
Leucocytes (4-11x10^9/L)	11.8x10^9/L	54.5x10^9/L
Neutrophils (2.15-7.68x10^9/L)	3.42x10^9/L	5.23x10^9/L
Lymphocytes (1.01-5.20x10^9/L)	8.8x10^9/L	45.9x10^9/L
Monocytes (0.19-0.96x10^9/L)	0.33x10^9/L	2,78x10^9/L
Eosinophils (0.024-0.51x10^9/L)	0.17x10^9/L	0.22x10^9/L
Basophils (0.0-0.16x10^9/L)	0.04x10^9/L	0.33x10^9/L
Platelets (150000-4000/uL)	253000/uL	223000/uL
Urea (19-49 mg/dL)	27 mg/dL	53 mg/dL
Creatinine (0.6-1.2 mg/dL)	1.0 mg/dL	1.1 mg/dL
Aspartate aminotransferase (12-40 U/L)	12 U/L	14 U/L
Alanine aminotransferase (7-40 U/L)	8 U/L	9 U/L
Total bilirubin (0.3-1.2 mg/dL)	0.2 mg/dL	0,2 mg/dL
Lactate dehydrogenase (135-225 U/L)	200 U/L	215 U/L
Sedimentation rate (<15 mm/1ªh)	12 mm/1ªh	73 mm/1ªh
C-reactive protein (<0.5 mg/dL)	0.7 mg/dL	5.9 mg/dL
Beta-2 microglobulin (0.8-2.2 ug/mL)	3 ug/mL	4.9 ug/mL
Hepatitis B surface antigen	0.44 (non-reactive)	-
Hepatitis B surface antibody	273 (reactive)	-
Antibody to hepatitis B core antigen	0.15 (reactive)	-
Hepatitis B virus DNA quantification	Undetectable	Undetectable

After one year of follow-up, the patient reported the appearance of multiple embossed non-pruritic erythematous skin lesions with irregular edges on the upper and lower limbs, converging in a cordonal path. The largest lesion was approximately 5 centimeters (cm) in diameter. Smaller papulonodular lesions were observed on the ankles. Of note, there was a lack of sensitivity to touch in such lesions. Skin biopsy revealed reticular dermis with perivascular, periadnexal, and perineural lymphohistiocytic infiltrate, presence of plasmocytes, rare polymorphonuclear eosinophils, and multiple histiocytes of eosinophilic granular cytoplasm, features suggestive of lepromatous leprosy. Nasal test detected acid-fast bacilli. When questioned, the patient reported a previous diagnosis of leprosy in his father; he also mentioned that, in Brazil, they used to hunt armadillos and eat their meat. Therefore, the skin lesions described and the histopathologic results, in the aforementioned epidemiological context, allowed the diagnosis of leprosy.

After the exclusion of glucose-6-phosphate dehydrogenase deficiency, a multidrug treatment (MDT) with dapsone, rifampicin, and clofazimine was initiated. Shortly thereafter, the patient developed new flat erythematous lesions with associated pain and heat, which were interpreted as type 1 reactions, showing good evolution with prednisolone 60 milligrams (mg) per day. Antibiotic therapy was continued throughout the reactional episode. Later, during the course of treatment, he developed hemolytic anemia probably related to dapsone. The drug was suspended and later reintroduced at a lower dose after hemoglobin recovery. He completed 24 months of triple treatment, with resolution of the skin lesions.

**Figure 1:**
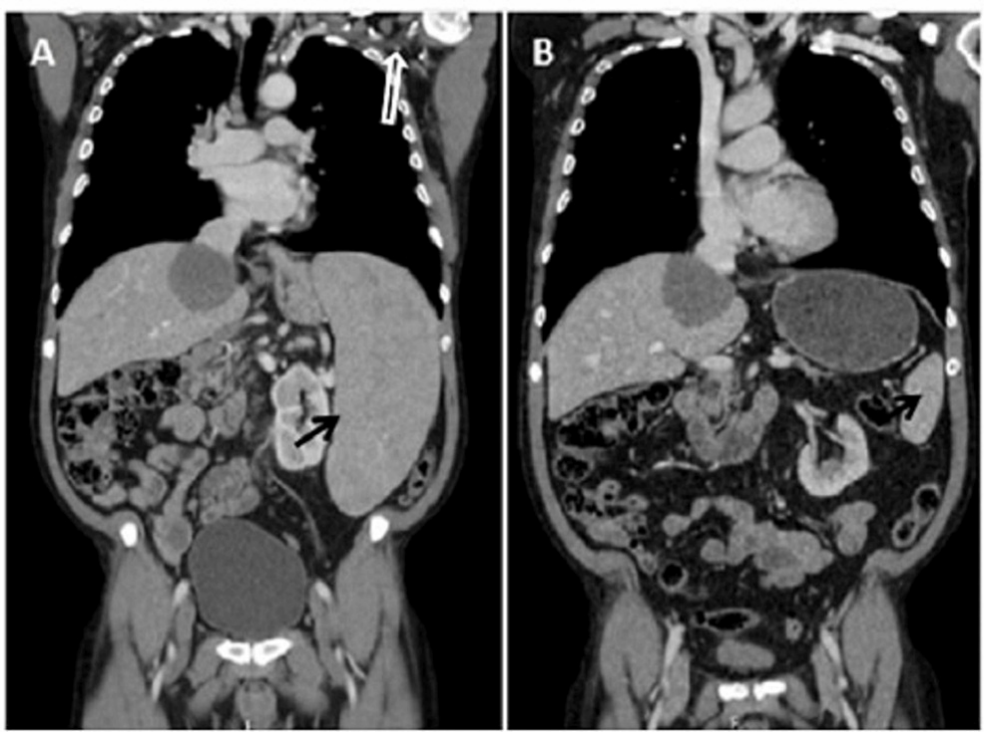
Thoraco-abdominopelvic CT scan (coronal plane comparison) A - at diagnosis; B - after R-CHOP chemoimmunotherapy. The black arrow highlights the enlarged spleen pre-treatment (A) and the normalization of its size after six cycles of chemotherapy (B). In the white arrow, it is possible to observe left axillary lymphadenopathies, absent after treatment.

After 38 months of the leprosy diagnosis, there was a gradual increase in lymphocyte count, without cytopenias or relevant biochemical alterations. Despite the rising lymphocytosis, he remained asymptomatic, without new findings on physical examination. However, after a three-year period of symptom-free status, the patient started experiencing early satiety and frequent night sweats. Physical examination revealed bilateral cervical and supraclavicular lymphadenopathies measuring 1 to 1.5 cm, as well as splenomegaly 6 cm below the left costal margin. Laboratory investigations showed increasing lymphocytosis 45.9x10^9/L and, for the first time, anemia (Hb 10.9 g/dL) (Table 1). A thoraco-abdominopelvic CT scan demonstrated two hepatic nodules suggestive of angiomas, peri- and infra-centimetric mediastinal and axillary lymphadenopathies, along with markedly enlarged homogeneous splenomegaly measuring 20 cm (Figure 1). Peripheral blood flow cytometry revealed that 93% of lymphocytes were small-B lymphocytes with the same abnormal immunophenotypic profile as observed in the flow study conducted 7 years prior. Additionally, a peripheral blood karyotype analysis demonstrated a translocation between chromosomes 11 and 14 in six out of 50 metaphases analyzed (46,XY,t(11;14)(q13;q32)(6)/46,XY(44)). Based on these clinical, laboratory, and imaging findings, the patient was diagnosed with mantle cell lymphoma, and treatment initiation was warranted.

The patient underwent an infectious disease consultation, which involved an extensive microbiological screening and a nasal test to detect acid-fast bacilli of the *Mycobacterium leprae* complex. Considering the finding of a past hepatitis B virus (HBV) infection and the increased risk of reactivation, entecavir 0.5 mg was initiated. The nasal test yielded negative results and prophylaxis for leprosy reactivation was not initiated, as there was no information to support its use in the available guidelines.

A chemoimmunotherapy protocol that consisted of R-CHOP induction (rituximab, cyclophosphamide, doxorubicin, vincristine, and prednisone) was then initiated. After six cycles of treatment, there was a great decrease in splenomegaly (13 cm) (Figure 1).

**Figure 2:**
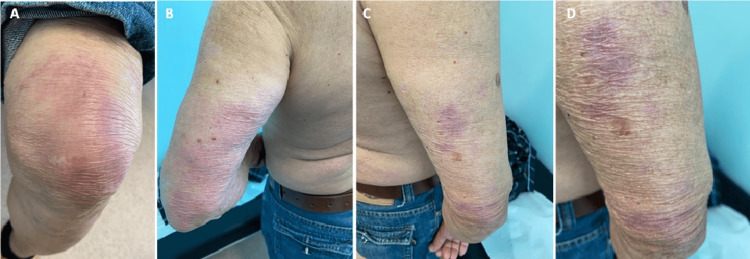
Erythematous skin lesions observed. A - lower right limb; B - upper left limb; C - upper right limb; D - close-up photography of the lesions on the upper right limb.

The patient refused to undergo autologous hematopoietic stem cell transplantation and thus proceeded with maintenance rituximab every 2 months, aiming for a total of 3 years of treatment. Prior to the second rituximab administration, new erythematous skin lesions were observed on the upper and lower limbs (Figure 2), accompanied with frequent pain in the left lower limb reported by the patient. A subsequent skin biopsy showed evidence of peri-vascular, periadnexal, and peri-neural lymphohistiocytic inflammatory infiltrates, along with focal non-necrotizing epithelioid granulomas and rare multinucleated giant cells. These findings were suggestive of borderline tuberculoid leprosy, confirming the occurrence of clinical relapse.

A new treatment regimen comprising rifampicin, clofazimine, and minocycline was initiated, with progressive improvement of the lesions and resolution within two years.

## Discussion

Leprosy is a chronic infectious disease caused by *Mycobacterium leprae* and *Mycobacterium lepromatosis*, which belong to the *Mycobacterium leprae* complex [1,5]. Although considered a disease of antiquity, leprosy continues to prevail, especially in tropical and subtropical regions, where it is mainly concentrated [2,6]. Approximately 80% of all new cases occur in India, Brazil, and Indonesia [4,7].

The transmission pathways of *Mycobacterium leprae* are not yet fully understood. There is strong evidence indicating a higher risk for individuals who have close contact with leprosy patients, most likely through infectious aerosols and skin-to-skin contact [3,5]. Furthermore, certain animals, such as armadillos and red squirrels, appear to be natural hosts and reservoirs for *M. leprae*, contributing to its transmission [5]. However, only 5% of individuals who come into contact with the bacillus develop the disease [3,5], suggesting that host immunity plays an important role in disease progression and control. The incubation time is variable, ranging from 2 to 20 years or longer [4]. In the case presented, the patient had a history of close contact with someone affected by leprosy and had also engaged in hunting armadillos, raising suspicion of leprosy. Interestingly, there was a 14-year interval between the onset of symptoms and the patient's last visit to Brazil. Although leprosy is mostly confined to tropical and subtropical regions, the long incubation period of this disease combined with the global flow of migrants, make it imperative that healthcare professionals do not overlook the possibility of this diagnosis.

Clinical presentation is determined by the host’s immune response to the infection and bacterial load, varying from paucibacillary (PB) forms with few bacilli to multibacillary (MB) forms with a high bacillary load [2]. The disease is classified into: polar tuberculoid leprosy, characterized by a strong cell-mediated immune response and few lesions with low or undetectable mycobacteria; and lepromatous leprosy, characterized by an ineffective immune response and presence of large amounts of bacilli, with patients developing diffuse and nodular skin lesions with hypoesthesia, and peripheral nerves are also affected, including muscle weakness and deformities of the extremities. Despite this division, the disease exists along a spectrum and patients can exhibit different forms of leprosy over time. In this particular case, the patient initially presented with clinical features consistent with lepromatous leprosy, which were confirmed by biopsy; however, during relapse, the disease exhibited characteristics of borderline tuberculoid leprosy.

Early diagnosis and complete treatment with multidrug therapy (MDT) remain the key strategies for reducing the disease burden of leprosy, but the recommended duration has evolved over time [4]. Initially, the minimum treatment duration was 24 months. However, current WHO guidelines recommend a 6-month regimen of rifampicin and dapsone for PB leprosy and a 12-month regimen of rifampicin, clofazimine and dapsone for MB leprosy. Some experts suggest the above triple regimen for all leprosy patients, with a treatment duration of 6 months for PB leprosy and 12 months for MB leprosy. Treatment for resistant leprosy should involve at least two second-line drugs (such as clarithromycin, minocycline, or a quinolone) in combination with daily clofazimine for 6 months, followed by clofazimine plus one of these drugs for an additional 18 months.

In the case presented, the patient completed a 24-month course of triple therapy, with treatment extended due to the temporary interruption of dapsone. Despite strict therapeutic adherence, the patient experienced a relapse of leprosy four years after completing antibiotic therapy, coinciding with the second administration of maintenance rituximab. Leprosy relapse occurs through the persistence of “hibernating” bacilli and is defined as patients presenting new signs of active disease after achieving a supposed cure with adequate treatment [8,9]. The risk of relapse is very low after completion of MDT, both for paucibacillary and multibacillary forms, and this is at least 10 times lower than with dapsone monotherapy [8]. WHO has estimated a risk of relapse of 0.77% for multibacillary disease and 1.07% for paucibacillary patients nine years after stopping MDT [8,10]. Predisposing factors for relapse include inadequate or irregular therapy, monotherapy, low health literacy and socioeconomic status, and male gender [10]. The role of rituximab or other chemotherapeutic/immunosuppressant agents in leprosy relapse remains uncertain and requires further investigation.

Rituximab is a chimeric monoclonal antibody targeting the B-cell antigen CD20, approved for the treatment of various malignant hematologic and rheumatologic disorders [11,12], that induces a rapid depletion of pre-B and mature B-cells, which can remain at low or undetectable levels for 2-6 months before gradually returning to pre-treatment levels, usually within 12 months. In addition to its effect on B-cells, rituximab has been shown to influence T-cell immunity and may predispose individuals to opportunistic infections [13].

Several studies suggest that peripheral B-cells play a role in the host defense against mycobacteria. In the context of rheumatoid arthritis, rituximab clinical trials excluded patients with a history suggestive of tuberculosis, and no cases of tuberculosis reactivation were reported, despite most patients having previously received TNF inhibitors. Infection with other mycobacteria has rarely been associated with rituximab - bacteremia caused by *Mycobacterium wolinskyi*, *Mycobacterium avium*, *Mycobacterium avium* pleuritis, and disseminated *Mycobacterium kansasii* infections have been described [13]. Few cases of leprosy have been reported in patients using immunosuppressors [14]. However, to our knowledge, there is no strong data to support an association between leprosy and rituximab. As such, there are no specific recommendations for leprosy prophylaxis in immunosuppressed patients. 

Mantle cell lymphoma (MCL) stands as an aggressive and rare form of B-cell non-Hodgkin lymphoma. Typically, it necessitates induction chemoimmunotherapy followed by autologous hematopoietic stem cell transplantation, particularly in younger and fit patients [15]. Recent studies have brought to light two variants of this disease: the classic variant and the indolent variant. The latter constitutes up to 30% of cases and is characterized by a leukemic presentation, SOX11 negativity, and a low proliferation index [16]. The clinical presentation of MCL varies significantly. It is conjectured that the indolent variant of this lymphoma can be effectively managed through observation alone, with reports indicating observation periods lasting up to 21 years from the initial presentation. This stands in stark contrast to classic MCL, where urgent chemoimmunotherapy is imperative [15, 16]. In the case presented, the definitive diagnosis of MCL was made more than 7 years after the identification of a CD5+ B lymphoproliferative disorder with phenotypic features that did not suggest B-CLL (B-cell chronic lymphocytic leukemia). The diagnosis was confirmed by the detection of t(11;14)(q13;32) translocation through conventional karyotyping of peripheral blood, that was carried out when the patient developed progression of splenomegaly and constitutional symptoms. Despite the patient being 67 years old and having no significant comorbidities, a chemoimmunotherapy regimen consisting of six cycles of R-CHOP followed by rituximab every two months for three years was chosen, due to the patient's refusal to undergo autologous hematopoietic stem cell transplantation.

Infectious agents can interact in complex ways to promote tumor development and progression. As a note, while an increased risk of developing mantle cell lymphoma has been described in patients with *Borrelia burgdorferi* and* Coxiella burnetii* infections [17], no studies suggest an association between leprosy and a higher risk of developing lymphoproliferative diseases.

## Conclusions

Although leprosy is mostly confined to tropical and subtropical regions, the long incubation period of this disease combined with the global flow of migrants, make it imperative that health professionals do not overlook the possibility of this diagnosis. The appearance of loss of sensation in pale or reddish skin lesions combined with a suggestive epidemiological context should alert the clinician to this diagnosis. Despite being rare, leprosy relapses are possible even after a few decades. The question of whether antibiotic prophylaxis should be considered in immunosuppressed patients undergoing immunochemotherapy for malignant diseases remains to be answered.
